# The Attitudes and Practices of General Practitioners about the Use of Chaperones in Melbourne, Australia

**DOI:** 10.1155/2012/768461

**Published:** 2012-08-17

**Authors:** Oliver van Hecke, Kay M. Jones

**Affiliations:** Department of General Practice, Monash University, Melbourne, VIC 3168, Australia

## Abstract

*Introduction*. To consider the use of medical chaperones during certain clinical examinations is important whether one practises as a specialist, nurse, medical student, or generalist. Chaperones have been used by doctors conducting intimate examinations for many years but their true extent remains largely unknown. Until recently, there was no national guidance in Australia. *Aim*. To explore the attitudes and practices of general practitioners (GP) regarding their use of chaperones in urban Melbourne, Australia. *Method*. Qualitative two focus groups involving seventeen GPs from two locations. Discussions were audio-taped, transcribed verbatim and analysed. *Results.* Common themes and subthemes emerged which were grouped into three main areas: (a) practitioner-related, (b) patient-related and (c) practice related. *Discussion*. This is the first study from an Australian primary care perspective to gauge the attitudes and experiences of GPs on their use of chaperones. It will provide vital information to inform the next step of extending this research to a national GP audience. From an international perspective, this study provides an excellent template for other primary care clinicians to conduct research in this important field of doctor-patient relationship.

## 1. Introduction

Medical chaperones are people, often health professionals that act as third-party observers during certain clinical examinations (most often intimate examinations), either at the request of the patient or because of the doctor's clinical judgement. The issue of medical chaperones is important whether one practises as a specialist, nurse, medical student, or generalist. This is repeatedly highlighted by newspapers and medical magazines where doctors (or nurses) have acted inappropriately. It often has life-changing repercussions for one or both parties [1, 2].

Although chaperones have been used by doctors conducting intimate examinations for many years, there seems to be no uniformity as to how and when chaperones should be used varying considerably between countries [3]. In some countries such as New Zealand and the United Kingdom, chaperones are used routinely [4, 5]. In other countries, such as Saudi Arabia, there is the potential influence of culture and religion [6], but in other countries, the use of chaperones remains largely unknown.

In Australia, there are no publications about the use of medical chaperones that relate to primary care where the vast majority of medical consultations occur. There is limited guidance from the Royal College of Australian General Practitioners (RACGP) [7]. In fact, the college raises the question about appropriateness and feasibility of the reported outcomes in general practice in Australia as most of the relevant research has been conducted overseas. It is thought that use of chaperones may be inherently different in Australia; in part due to a wide variation in clinician, patient, and public attitude and diverse cultural and religious influences. This qualitative study is the first attempt to try and gauge the attitudes and experiences of GPs (general practitioners) with Australia in respect to the use of chaperones.

**Aim**

To explore the attitudes and practices of a cohort of GPs practising in Melbourne, Australia, regarding the use of chaperones in general practice.

## 2. Method

### 2.1. Qualitative

 Data were collected during two focus groups.

#### 2.1.1. Recruitment

Seventeen GPs were recruited from two locations with different work patterns; ten volunteered in response to a poster advertisement at the Melbourne Metropolitan Deputising Service (MMDS), located in inner Melbourne. MMDS provides after-hours services throughout metropolitan Melbourne. Seven GPs volunteered in response to a seminar advertisement at Monash University Department of General Practice (MU) and also work in general practice, providing mostly daytime medical care. The number of participants in the focus groups was restricted to a maximum of 10 in each focus group.

#### 2.1.2. Data Collection

Data were collected using the semistructured interview schedule that was developed using the available literature. The schedule included prompts regarding the GPs' experiences of using a chaperone, possible influences and importance of using a chaperone, and any barriers. The two focus groups were held in April 2011, at MDDS (approximately 120 minutes) and Monash University, Department of General Practice (approximately 90 minutes). The focus groups were audio-taped and transcribed verbatim.

#### 2.1.3. Data Analysis

Data were analysed according to the Framework Method [8] which involves a process of becoming familiar with the content of the data, identifying recurring words and themes, and interpreting the themes to understand participants' perspectives [8–10]. Data were analysed independently by the two investigators; when there was a difference of opinion, the issues were discussed and agreement was reached. To ensure privacy and anonymity, statements are reported as either (MMDS) or (MU).

#### 2.1.4. Ethics

This study was approved by the Monash University Human Research Ethics committee (MUHREC). Written informed consent was obtained from each participant.

## 3. Findings

### 3.1. Demographics

#### 3.1.1. MMDS

 The ten GPs comprised seven males and three females, of those, six males and two females were born and trained overseas; the other male and female were born overseas and trained in Australia. One was less than 40 years and nine were aged 40+. Although currently living and working in Australia, all indicated a strong connection with what they described as their “country of origin.”

#### 3.1.2. MU

 The seven GPs comprised three males and four females, of those, one female was born and trained overseas and indicated a strong connection to her country of origin. The remainder were Australian born and trained, and described their connection as “Australian.” All were aged 40+.

The findings and discussion are reported under the three main themes and sub-themes that emerged during the data analysis (see Table 1). The three main themes are grouped into those related to:

- practitioner,
- patient,
- practice (workplace).

**(a) Practitioner-Related**

*Background and Training of the GP.*  Opinion ranged about what may influence GPs. Some acknowledged that training and background of the GP as well as familiarity with Australian medical environment may influence the use of chaperones. Others felt that cultural or religious background may be potentially more significant than the country of training.

*“*[medical]* students from a different cultural background, being trained here *[in Australia]* … you might find their cultural background may be more significant than their training, particularly around beliefs and attitudes (MU).”*

*“… or is it about the ethnicity of your patient, or the religious background or the age of your patient (MU)?”*

**Situational Awareness and Professional Judgement**

Participants highlighted the importance of situational awareness before undertaking any examination in particular nonintimate examinations that might be misconstrued by the patient.

*“I would really question a patient who wanted a chaperone for an eye exam … in the sense that none of our peers would probably provide a chaperone for an eye exam? (MU).”*

*“I would never have thought of a back examination or manipulation as intimate (MU).”*

*“ … *[during a]* home visit, we do not often see a patient *[for]* a general examination like a PV *[vaginal)* or PR *[rectal]* examination (MMDS).”*

**Familiarity with Patient**

Suggesting a chaperone might be perceived as introducing the idea that the GP is untrustworthy into a previously good relationship between the GP and patient. Some participants argued that a chaperone may be unnecessary in certain circumstances as patients may often choose the doctor they see and trust.

*“ … if you've known the patient for a long time, you've delivered *[their]* baby and stitched up an episiotomy, you have a fairly intimate relationship with that person as opposed to the person who's just walked in off the street (MU).”*

**Doctor-Patient Relationship**

The groups acknowledge the importance of the doctor-patient relationship and the inherent power differential between doctor and patient and how this can vary between doctor and patient of dissimilar background. The artificial or formal nature of using a chaperone was raised, with the potential of distorting the consultation process and possibly impacting on doctor-patient relationship.

*“Sometimes I don't feel comfortable examining a female patient, I don't know why. You can't impose a nurse every time you examine one (MU).”*

* “I had a chance to work with the very senior GP who was an examiner for fellowship examination as well. And he said he never uses a chaperone and that it is right not to. *[He added]* … a patient that has walked into his room for a pap smear, knows he's a male doctor and she has to trust me … but even if they were to ask him *[for a chaperone]* he would tell them he doesn't use a chaperone (MMDS)”.*

**(b) Patient-Related**

*Ethnic, Cultural and Religious Background of Patient*.  GPs spoke of their personal experience in dealing with intimate examinations when patients are from a different background to them; they highlighted the impact this may have on how the examination is conducted, and the offer and use of a chaperone.

*“ … I would have young women [come] straight off the street and ask for a smear. I was obviously more uncomfortable in some ways than they were, but they were fine with it (MU)”.*

*“I've been in a situation with a Muslim woman, I end up taking vitals, assessing if she is broadly well or unwell. I then have to say you'll have to see a female GP. Even with the husband there you cannot go any further (MMDS).”*

**Patient Embarrassment**

More subtle reasons influencing the use of a chaperone include patient embarrassment and lost opportunities to discuss intimate issues in the presence of a third party.

*“I think sometimes you offer people *[a chaperone]* and they say it's bad enough to have one person *[here]*, than to have someone else as well (MMDS).”*

**Patients' Lack of Awareness**

It is likely that patients attending a GP in Australia may not be familiar with the option of a chaperone. The groups highlighted that a chaperone policy may be an excellent example of the profession's need to communicate in language appropriate to the patient group.

* “Some practices may not raise the topic, […], maybe never even thought about it, never raised it with the doctors (MMDS).”*

* “Sometimes patients consent and then they find it *[the intimate examination]* difficult … even knowing what they're consenting to (MU).”*

**Privacy and Confidentiality**

Using chaperones in a small community or town highlighted the issue of privacy and confidentiality.

*“If you work in a rural area in a smaller practice, everybody knows everybody, no one wants the receptionist to come in and chaperone (MU).”*

**(c) Practice-Related**

*The Interpretation of the Term “Medical Chaperone.”*  One issue raised in both groups concerned the meaning of “medical chaperone”. Participants related that in most cases, a member of the practice staff would be considered as a medical chaperone. However, a chaperone in primary care may not necessarily have a health professional background. Also, unlike colleagues in the secondary care setting, GPs are sometimes required to attend patients at home or in a nursing home and are usually unaccompanied, particularly those working after-hours. On a home visit, the chaperone might be, for example, the GPs' driver or a medical student accompanying the GP.

* “It's a difficult situation in the home visits, because you are literally unprotected if you do not have a chaperone there and it's you versus the patients (MMDS).”*

* “I think in terms of general practice and out of hours, because we do home visits, we are at particular risk and it's about what is actually feasible because a chaperone might not always be feasible (MDDS).”*

**Expectations about Gynaecological Examinations**

The group highlighted that often gynaecological examinations were carried out solely by female GPs. One issue subsequently highlighted the need for male GPs to maintain their level of confidence, professionalism, and skills by continuing to perform intimate examinations.

*“A lot of female GPs [mainly do] the tears and smears (MU).”*

*“We are all trained to do medical examinations; if we do not do a gynae examination, breast examination, we won't know how to do a proper one (MMDS).”*

### 3.2. Chaperone Availability

The provision of a chaperone may not be feasible in all circumstances in general practice in Australia for a number of reasons, for example, during home visits or because of the geographical outlay of Australia. Apart from GPs practising as sole practitioners, particularly in the outlying regions, some urban practices have no practice nurses; others only have a practice nurse for limited hours per day.

*“Á place that has got a practice nurse and they come in once a week, it's totally different to one that's got a full time practice nurse (MU).”*

### 3.3. Economic Costs

Many participants echoed the economic implications of additional resources needed to implement chaperones but also raised issues such as keeping records about the offer and use of chaperones and the impact on the doctor-patient relationship.

*“A chaperone involves time, and therefore money in providing a chaperone, and it's not just the cost, it's the logistics as well (MU).”*

*“… it can be an issue in a GP partnership where one partner feels he should provide a chaperone and the other partner does not want to pay for it (MDDS).”*

### 3.4. Timing of Discussion and the Use of a Chaperone

GP participants related that some patients may not request a chaperone because they feel uncomfortable requesting a chaperone or might not know that such an option exists. GPs agreed that would be feasible for the clinic to arrange a chaperone if it was a scheduled procedure, for example, a Pap-smear. However, GPs also emphasised that offering a chaperone may be more useful in unanticipated intimate examinations.

* “The thing about chaperones it's not a regular event, it's fairly irregular, it's a bit like interpreters (MU).”*

**“[Patients]* do not necessarily expect an *[intimate]* examination. A lot of patients will feel intimidated. If you offered a chaperone *[they]* may be grateful for that (MU).”*

## 4. Discussion

One of the most important components of the doctor-patient relationship is trust and respect for a patient's autonomy, and these can be expressed in different ways [11]. The power differentials that exist between doctors and their patients can be subtle, as can the vulnerability patients may feel. Thus, using a chaperone is both an added layer of protection and acknowledgement of a patient's vulnerability [1, 4, 12, 13].

A search of the published literature worldwide found limited research about the use of chaperones overseas whether in primary care [5, 12, 14, 15] or hospital clinics [6, 16–19] about patients' awareness [13, 17, 20], protection for patients and/or doctors [3, 11, 21], or guidelines [22–24]. Similarly, little literature was found about the use of chaperones in Australia; results of two studies conducted in sexual health clinics [4, 25, 26], commentaries about doctors' misconduct [1], and risk management [27], as well as three documents providing medicolegal guidance [7, 28, 29]. We could not find any published research that pertains to the Australian general practice setting.

The main thrust of our findings highlights the main influences of the use of chaperones in this pilot group of Australian GPs (Table 1).

The group raised similar concerns and attitudes as international GP colleagues [14] but also added unique insight into local attitudes on the use of chaperones in general practice for example, the influence of diverse cultural influences from the Asia-Pacific region, the unique geographical setting of general practice in Australia and chaperone availability amongst others.

At the time of conducting this pilot study, the Medical Board of Australia (MBA) issued guidelines on sexual boundaries offering medical practitioners some clearer guidance [29]. While best practice may be for services to routinely offer a chaperone and record instances where an offer is declined, the provision of a chaperone may not be feasible in all circumstances—particularly in Australian general practice. Thus, flexible guidance is needed for general practice including doctors being urged to communicate more openly with patients about the use of chaperones [14]. For most patients, being reassured and given a choice of having the right to accept or decline the offer of a suitably qualified chaperone of their choice, takes precedence over the need for a chaperone [3, 4, 18].

## 5. Strengths and Limitations

This qualitative study is the first attempt to record the attitudes and experiences of GPs about their use of chaperones in general practice in Australia at a time when there was no formal guidance. While not all participants supported the idea of formal guidance, all agreed that further discussion and research from a wider audience are needed from an Australian perspective. From an international perspective, the use chaperones does not appear to have been explored, thus generally remains unknown, and what is known is limited to a handful of countries. This is odd since all primary care physicians will at some time in their career conduct an intimate examination. This provides an excellent template for other primary care clinicians to conduct research in this important field of doctor-patient relationship.

This pilot study made no attempt to recruit a truly representative sample of GPs practising in Australia. The study is limited to data from two focus groups and to GPs working in general practice and/or an after-hours medical deputising service in urban Melbourne. The views of other health-care workers and patients were not explored.

## 6. Implications for General Practice

This study is the first step in understanding the attitudes and experiences of GPs practising in Australia about the use of chaperones in general practice. There is a need to complement these views with a larger GP audience but also with patients who attend their general practitioner.

## Figures and Tables

**Table 1:** Summary of main themes and sub-themes.

Themes	Subcodes
(a) Practitioner-related	(i) GP background and training
	(ii) Situational awareness and professional judgement
	(iii) Familiarity with patient
	(iv) Doctor-patient relationship

(b) Patient-related	(i) Ethnic, cultural, and religiousbackground
	(ii) Patient embarrassment
	(iii) Patient's lack of awareness
	(iv) Privacy and confidentiality

(c) Practice-related	(i) Interpretation of “chaperone”
	(ii) Expectation of examination
	(iii) Chaperone availability
	(iv) Economic resources
	(v) Timing and the use of a chaperone
